# Blockade of Acid-Sensing Ion Channels Increases Urinary Bladder Capacity With or Without Intravesical Irritation in Mice

**DOI:** 10.3389/fphys.2020.592867

**Published:** 2020-10-26

**Authors:** Mitsuharu Yoshiyama, Hideki Kobayashi, Masayuki Takeda, Isao Araki

**Affiliations:** ^1^Department of Urology, Graduate School of Medicine, University of Yamanashi, Chuo, Japan; ^2^Shintotsuka Hospital, Yokohama, Japan; ^3^Kobayashi Urology Clinic, Kai, Japan; ^4^Kusatsu Public Health Center, Kusatsu, Japan

**Keywords:** A-317567, acetic acid, bladder pain syndrome, dorsal root ganglion, intravesical irritation, mechanosensation, nociception

## Abstract

We conducted this study to examine whether acid-sensing ion channels (ASICs) are involved in the modulation of urinary bladder activity with or without intravesical irritation induced by acetic acid. All *in vivo* evaluations were conducted during continuous infusion cystometry in decerebrated unanesthetized female mice. During cystometry with a pH 6.3 saline infusion, an i.p. injection of 30 μmol/kg A-317567 (a potent, non-amiloride ASIC blocker) increased the intercontraction interval (ICI) by 30% (*P* < 0.001), whereas vehicle injection had no effect. An intravesical acetic acid (pH 3.0) infusion induced bladder hyperactivity, with reductions in ICI and maximal voiding pressure (MVP) by 79% (*P* < 0.0001) and 29% (*P* < 0.001), respectively. A-317567 (30 μmol/kg i.p.) alleviated hyperreflexia by increasing the acid-shortened ICI by 76% (*P* < 0.001). This dose produced no effect on MVP under either intravesical pH condition. Further analysis in comparison with vehicle showed that the increase in ICI (or bladder capacity) by the drug was not dependent on bladder compliance. Meanwhile, intravesical perfusion of A-317567 (100 μM) had no effect on bladder activity during pH 6.0 saline infusion cystometry, and drug perfusion at neither 100 μM nor 1 mM produced any effects on bladder hyperreflexia during pH 3.0 acetic acid infusion cystometry. A-317567 has been suggested to display extremely poor penetrability into the central nervous system and thus to be a peripherally active blocker. Taken together, our results suggest that blockade of ASIC signal transduction increases bladder capacity under normal intravesical pH conditions and alleviates bladder hyperreflexia induced by intravesical acidification and that the site responsible for this action is likely to be the dorsal root ganglia.

## Introduction

Interstitial cystitis (IC)/bladder pain syndrome (BPS) is a chronic bladder healthcare issue comprising severe refractory bladder symptoms such as suprapubic pain and urinary frequency and urgency, without a specific identifiable cause (Yoshimura et al., 2002). The etiology of the disease is thought to be multifactorial and needs to be further explored. Remedies for IC/BPS include conservative treatments such as stress reduction (Nickel et al., 2010) and physical therapy (Bassaly et al., 2011), invasive therapy including hydrodistension (El-Hefnawy et al., 2015), intravesical application of chemicals (Peeker et al., 2000), and pharmacotherapy. Orally active drugs, including amitriptyline (Hanno and Wein, 1987), hydroxyzine (Theoharides and Sant, 1997), and cimetidine (Seshadri et al., 1994), can be given, although their effectiveness is inconclusive and controversial. Thus, further investigations for developing novel treatments to alleviate symptoms of IC/BPS are warranted.

Transient receptor potential vanilloid receptor 1 (TRPV1) has been suggested to be a candidate target in the treatment of IC/BPS (Liu et al., 2014; Grundy et al., 2018; Rosen et al., 2018). TRPV1 is an ion channel activated by capsaicin, protons (pH ≤ 6), heat (≥ 43°C) and endogenous ligands such as anandamide, and is widely expressed in peripheral organs, the peripheral nervous system, and the central nervous system of mammals (Caterina et al., 1997; Tominaga et al., 1998; Avelino et al., 2002; Avelino and Cruz, 2006). Previous studies have shown a possible involvement of TRPV1 in the modulation of irritated urinary bladder activity via signal transduction in the urothelium and via vesical afferent C-fiber transmission (Birder et al., 2002; Dinis et al., 2004; Wang et al., 2008), suggesting a potential therapeutic use of TRPV1 antagonists for IC/BPS. TRPV1 antagonists, however, have been found to significantly affect body temperature (Garami et al., 2020), contraindicating their clinical use and impeding further development of their applicability.

Acid-sensing ion channels (ASICs) are members of the amiloride-sensitive sodium channel superfamily, including the epithelial sodium channel/degenerin (ENaC/DEG), that play crucial roles in mechanosensation, chemosensation, and nociception (Kellenberger and Schild, 2002; Wemmie et al., 2006; Lingueglia, 2007). Mammals possess seven ASIC isoforms, ASIC1a, ASIC1b, ASIC2a, ASIC2b, ASIC3, ASIC4, and ASIC5, which are encoded by five genes (Accn1, Accn2, Accn3, Accn4, and Accn5). The ASIC1a, ASIC1b, ASIC2a, and ASIC3 subunits can combine to form homotrimeric or heterotrimeric channels that differ in their pH sensitivity and other pharmacological properties (Chen and Wong, 2013; Hanukoglu, 2017). ASIC2b is not activated by reduced pH and is non-functional alone but modulates channel activity when participating in heteromultimers. The functions of ASIC4 and ASIC5 are yet unknown.

A previous study revealed that the genes of the ASIC subunits ASIC1, ASIC2, and ASIC3 are largely expressed in mouse urinary bladder (mucosa and detrusor) and lumbosacral L6/S1 dorsal root ganglion (DRG) neurons innervating the bladder and that the amount of each ASIC-subunit gene is similar to or greater than that of the TRPV1 gene in mice (Kobayashi et al., 2009), suggesting the possibility that ASICs are involved in bladder mechanosensation and nociception via urothelial signal transduction and vesical afferent pathways. Thus, we conducted this study using an ASIC blocker to examine whether ASICs play a functional role in the control of urinary bladder activity with or without intravesical acidic irritation, especially during the bladder collection period.

The compound A-317567 (6-{2-[2-methyl-1-(propan-2-yl)-1,2,3,4-tetrahydroisoquinolin-7-yl]cyclopropyl}naphthalene-2-carboximidamide) used in this study is a non-amiloride blocker of ASICs (Dubé et al., 2005). In acutely dissociated adult rat DRG neurons, A-317567 was shown to produce a potent blocking of ASIC3 with an IC_50_ of 1.025 μM and to equipotently block the sustained phase of the ASIC3-like current, a biphasic current akin to that of cloned ASIC3 (Dubé et al., 2005). A-317567 was also shown to inhibit all pH 4.5-evoked ASIC currents with an IC_50_ ranging between 2 and 30 μM in a concentration-dependent fashion, depending on the activated current-type of ASIC. Thus, A-317567 can block ASIC1 and ASIC2 as well as ASIC3. In *in vivo* experiments, an intraperitoneal (i.p.) injection of A-317567 efficaciously exerted an analgesic effect on complete Freud’s adjuvant-induced inflammatory thermal hyperalgesia and postoperative pain caused by skin incision (Dubé et al., 2005). A-317567 is a small molecule with a molecular weight of 397.56; thus, it is suitable for both intravesical administration and systemic injection in the investigation of the *in vivo* roles of ASICs in micturition reflex pathways, including the urothelium, detrusor and nervous systems.

Preliminary results have been presented in an abstract (Yoshiyama et al., 2014).

## Materials and Methods

### Animal Preparation

Fifty-three female mice (C57BL/6, 12–13 weeks old; Charles River Laboratories, Yokohama, Japan) weighing 19.5–24.5 g were used in this study. We previously found that increases in micturition frequency in response to intravesical acidic irritation were much greater in females than males (Yoshiyama et al., 2008, 2010). Thus, females were chosen because they could facilitate evaluation of the expected drug effect on bladder hyperreflexia. The animals were housed under a 12:12-h light-dark cycle with controlled humidity and temperature. Standard pellet diet and water were available *ad libitum*. All animal procedures were reviewed and approved by the University of Yamanashi Institutional Animal Care and Use Committee. All efforts were made to minimize animal suffering and to reduce the number of animals used.

The animals were anesthetized with sevoflurane (2–3%) in oxygen (flow rate: 0.2 l/min) during surgery before decerebration. The trachea was cannulated with a polyethylene tube (PE-90; Clay-Adams, Parsippany, NJ) to facilitate respiration. The bladder was exposed by way of a midline abdominal incision. The bladder end of a polyethylene catheter (PE-50; Clay-Adams) was heated to create a collar and passed through a small incision at the apex of the bladder dome, and a suture was tightened around the collar of the catheter. The incised abdominal muscles as well as the incised skin were loosely sutured, and the catheter exited near the xiphoid process. For i.p. administration, PE-10 tubing was passed through a slit made near the xiphoid process, and the tip of the tube was placed at the right edge of the upper intra-abdominal region of the animals.

Precollicular decerebration was performed according to a previously published method (Sapru and Krieger, 1978) that included ligating both carotid arteries, followed by a midline incision of the head skin with a scalpel and removal of the skull and forebrain using a fine rongeur and a blunt spatula, respectively. Sevoflurane was then discontinued. After no further intracranial hemorrhage was visually detected, both lateral flaps of the incised head skin were sutured together. Experiments were started 2 h after the decerebration and conducted under unanesthetized conditions (Yoshiyama et al., 2008, 2010, 2015; Kira et al., 2017).

### Cystometric Recordings

**FIGURE 1:**
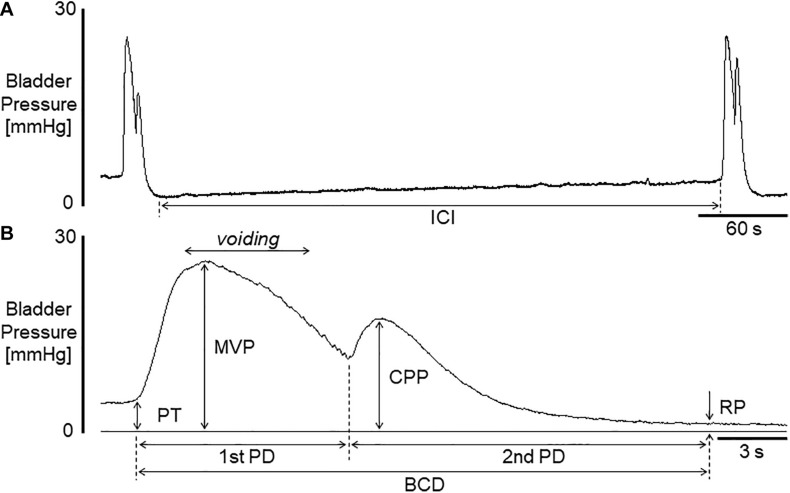
**(A)** Representative voiding cycle during cystometry in a mouse. **(B)** A bladder contraction extended from that on left in **(A)**. Voiding occurred during the period indicated in **(B)**. ICI, intercontraction interval; BCD, bladder contraction duration; 1st PD, 1st phase contraction duration; 2nd PD, 2nd phase contraction duration; PT, pressure threshold for inducing micturition contraction; MVP, maximal voiding pressure; CPP, closing peak pressure; RP, resting pressure. Bladder compliance (BCP) was calculated as infusion rate × (ICI/60)/(PT - previous RP).

All cystometric evaluations were conducted under unanesthetized conditions in decerebrated mice to assess ‘reflex micturition’. Pros and cons when using this animal model have been described elsewhere (Fraser et al., 2020). Saline (pH 6.3) was infused into the bladder for 2 h before baseline values were measured. Bladder activity was monitored by way of a cystometry catheter connected to a pressure transducer. Cystometric recordings were performed by continuously infusing physiological saline (30 μl/min) at room temperature into the bladder to elicit repetitive voids, which allowed data collection for a large number of voiding cycles (Maggi et al., 1986; Fraser et al., 2020). As shown in Figure 1, cystometric parameters measured were: pressure threshold (PT; mmHg), the intraluminal pressure required to induce a voiding contraction; maximal voiding pressure (MVP; mmHg), the peak intraluminal pressure during voiding; closing peak pressure (CPP; mmHg), the peak pressure during the postvoiding phase of a bladder contraction; resting pressure (RP; mmHg), the lowest pressure immediately after a voiding contraction; and intercontraction interval (ICI; s), the time lag between two voiding cycles. An additional parameter, the bladder contraction duration (BCD; s) is the sum of the first-phase duration (1st PD; s) and the second-phase duration (2nd PD; s). In general, voiding occurs during part of the first phase of bladder contraction, and the second phase of the contraction occurs in the bladder-filling period (Yoshiyama et al., 2008). We also evaluated bladder compliance (BCP; μl/mmHg), which was calculated as the ratio of infused volume to the pressure difference between time points of RP and the following PT, i.e., (infusion rate x ICI/60) (in μl)/(PT - previous RP) (in mmHg). The significance of evaluating these parameters was stated in previous reports (Yoshiyama et al., 2008, 2010; Kira et al., 2017). Acetic acid infusion into the bladder has been widely used as a model for acute irritation of the lower urinary tract (Yoshiyama et al., 1993b, 2008, 2010). The effects of diluted acetic acid (pH 3.0) intravesically infused for 1 h on bladder activity were examined following the baseline infusion of saline.

### Drugs

Sevoflurane (Maruishi Pharmaceutical, Osaka, Japan), acetic acid (99.7%; Nacalai Tesque, Kyoto, Japan), and ethanol (Wako Pure Chemical Industries, Osaka, Japan) were used in the present study. A-317567 dihydrochloride was discretely synthesized in a private institute. A-317567 was dissolved in physiological saline and adjusted to pH 2.8 and pH 6.0 with 1 N hydrochloride (HCl) for i.p. injection (100 mM) and intravesical perfusion (100 μM), respectively. A-317567 was also dissolved in dilute acetic acid (pH 3.0) for intravesical perfusion (100 μM and 1 mM) to examine the effect of the drug on bladder hyperactivity during irritation of the lower urinary tract. The dose for i.p. injection was 30 μmol/kg; this dose was chosen because it had been shown to produce significant analgesic effects in a previous study (Dubé et al., 2005). Drug or vehicle was administered via an i.p. catheter (300 μl/kg), followed by 8 μl air injection to empty the tube. Then the effects of A-317567 were compared with those of vehicle.

### Data Analysis and Statistics

Three consecutive storage periods and voiding contractions during intravesical saline (pH 6.3) infusion immediately before i.p. injection of A-317567 or vehicle (pH 2.8 solution) or start of A-317567 or vehicle (pH 6.0 or pH 3.0 solution) perfusion were evaluated as the baseline. Changes in cystometric variables during intravesical infusion of saline (pH 6.0) or acetic acid (pH 3.0) were compared between A-317567 and vehicle. All values are expressed as the means ± S.E.M.s Statistical analysis was performed using unpaired t tests or two-way repeated measures ANOVA followed by Sidak’s multiple comparisons test, if applicable. For all analyses, *P* < 0.05 was considered significant.

## Results

Basal cystometric variables before drug administration or intravesical perfusion of the chemical irritant were measured during normal saline (pH 6.3) infusion cystometry, as shown in Table 1.

**TABLE 1:** Variables measured during continuous infusion cystometry in female mice.

PT (mmHg)	MVP (mmHg)	CPP (mmHg)	RP (mmHg)	1st PD (s)	2nd PD (s)	BCD (s)	ICI (s)	BCP (μl/mmHg)
5.2 ± 0.2	23.2 ± 0.4	13.5 ± 0.6	1.5 ± 0.1	10.2 ± 0.5	16.4 ± 0.5	26.5 ± 0.8	322.4 ± 12.7	48.4 ± 2.3

*Values are means ± SEM for 53 mice. PT, pressure threshold for inducing micturition contraction; MVP, maximal voiding pressure; CPP, closing peak pressure; RP, resting pressure; 1st PD, 1st phase duration of bladder contraction; 2nd PD, 2nd phase duration of bladder contraction; BCD, bladder contraction duration; ICI, intercontraction interval; BCP, bladder compliance.*

### Intraperitoneal Injection of A-317567

**FIGURE 2:**
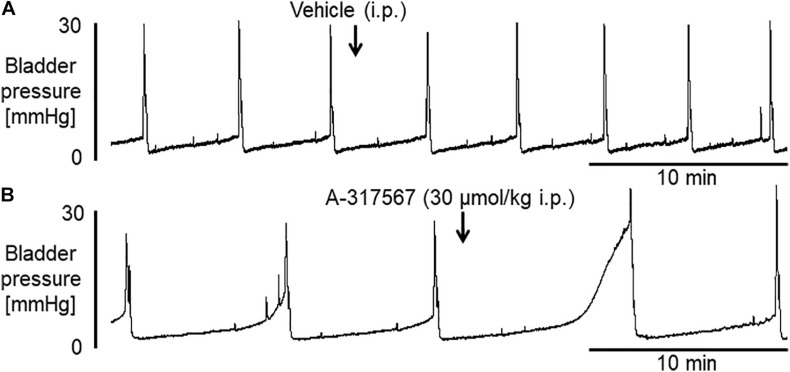
Bladder activity was induced in female mice during continuous infusion cystometry (30 μl/min) with saline (pH 6.3). These recordings are from 2 different mice. **(A)** I.p. injection of vehicle produced no effect on bladder activity. **(B)** A-317567 (30 μmol/kg i.p.) overtly increased ICI and PT. An increase in MVP was evident in this mouse, although the effect of the drug on MVP varied among the different mice. The effects on these affected variables disappeared within 20 min.

**FIGURE 3:**
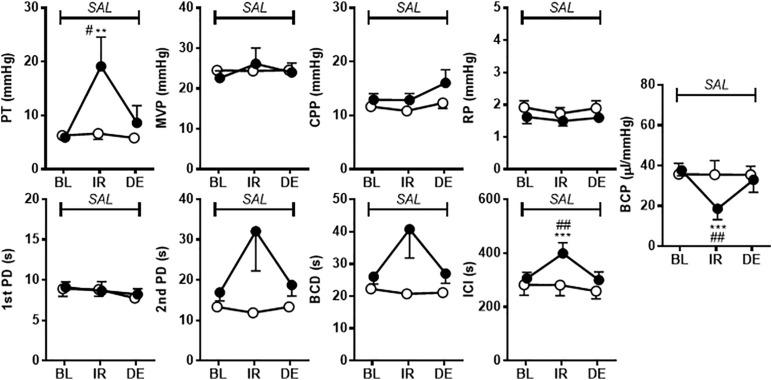
The effects of i.p. administration of A-317567 (30 μmol/kg, •, *n* = 6) or vehicle (∘, *n* = 6) on bladder activity during saline (pH 6.3) infusion cystometry were evaluated according to the presented cystometry parameters. Statistical analysis by two-way repeated measures ANOVA revealed that, in comparison with vehicle, A-317567 significantly elevated PT, prolonged ICI and decreased BCP (^#^*P* < 0.05 and ^##^*P* < 0.01), but it did not change any other cystometry variables. The differences from BL to IR are indicated as **P < 0.01 and ***P < 0.001 by *post hoc* Sidak’s multiple comparisons test. SAL, saline (pH 6.3) infusion; BL, baseline; IR, immediate response to the i.p. injection; DE, disappearance of effect, which was evaluated 20 min after the injection in the series of experiments.

Figure 2 shows the effects of i.p. injection of vehicle or A-317567 (30 μmol/kg) on bladder activity during pH 6.3 saline infusion cystometry. Even after A-317567 injection, as the infused volume in the bladder increased, bladder pressure gradually increased following the same trend as the change in intravesical pressure from baseline (before drug injection). However, after the infused volume in the bladder was greater than the volume at baseline, the intravesical pressure increased exponentially (Figure 2). Delivery of vehicle via i.p. injection had no effect on urinary bladder activity during saline infusion cystometry, whereas I,p. injection of A-317567 (30 μmol/kg) increased the ICI by 30% (range: 12 to 61%), elevated the PT by 206% (range: 24 to 468%) and decreased the BCP by 53% (range: 9 to 83%), all of which were significantly different from of the levels upon vehicle injection (Figure 3). The changes induced by A-317567 reverted to the baseline conditions within 20 min in all animals (*n* = 6). No differences in MVP, CPP, RP, 1st PD, 2nd PD, or BCD were found between the A-317567 and vehicle groups (Figure 3). Given the variable changes induced by A-317567 in the 2nd PD that resulted in the variable BCD values, neither parameter was significantly different from the corresponding parameter in the vehicle group (*P* = 0.15 and *P* = 0.13, respectively, by two-way repeated measures ANOVA).

**FIGURE 4:**
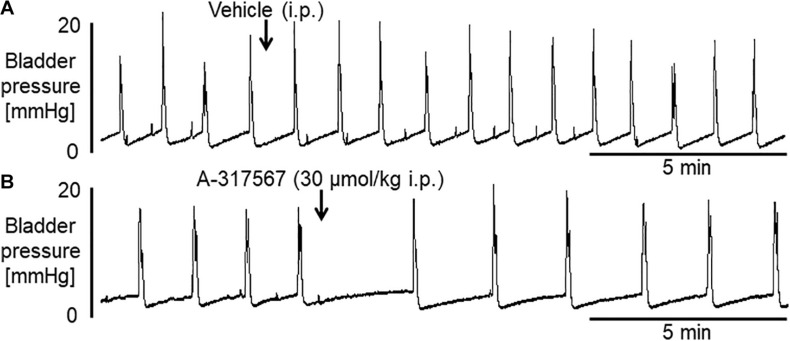
Frequent voidings were elicited in mice during continuous infusion cystometry (30 μl/min) with diluted acetic acid (pH 3.0). These recordings are from 2 different mice. **(A)** I.p. injection of vehicle produced little effect on the hyperactive bladder. **(B)** A-317567 (i.p.) alleviated the acetic acid-induced bladder hyperreflexia. The effect lasted for approximately 10 min.

**FIGURE 5:**
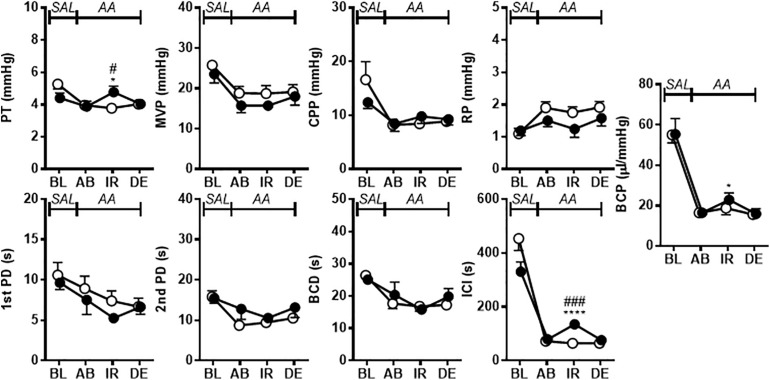
The effects of i.p. administration of A-317567 (30 μmol/kg, •, *n* = 6) or vehicle (∘, *n* = 6) on bladder activity during diluted acetic acid (pH 3.0) infusion cystometry were evaluated according to the presented cystometry parameters. Statistical analysis by two-way repeated measures ANOVA revealed that, in comparison with vehicle, A-317567 significantly elevated PT and increased ICI (^#^*P* < 0.05 and ^###^*P* < 0.001), but it did not change any other cystometry variables. The differences from AB to IR are indicated as *P < 0.05 and ****P < 0.0001 by *post hoc* Sidak’s multiple comparisons test. SAL, saline (pH 6.3) infusion; AA, acetic acid (pH 3.0) infusion; BL, baseline; AB, acidic baseline; IR, immediate response to the i.p. injection; DE, disappearance of effect, which was evaluated 10 min after the injection in the series of experiments.

Figure 4 shows the effects of i.p. injection of vehicle or A-317567 on bladder activity during pH 3.0 diluted acetic acid infusion cystometry. Changing the intravesical infusion solution from pH 6.3 saline to pH 3.0 diluted acetic acid greatly affected the values of the cystometric variables except for the 1st PD (Table 2) and induced urinary bladder hyperreflexia (Figure 4). Even after drug injection, as the infused volume in the bladder increased to the basal volume, bladder pressure gradually increased following the same trend as the change in intravesical pressure from baseline (Figure 4). Delivery of A-317567 (30 μmol/kg) i.p. injection significantly increased the acid-reduced ICI and PT by 76% (range: 32 to 139%) and 25% (range: 1 to 52%), respectively, all of which were significantly different from of the levels upon vehicle injection (Figure 5). Vehicle treatment had no effects on any of the evaluated cystometric variables. The effects of A-317567 lasted for 10–15 min in all animals (*n* = 6). Compared with those of vehicle, A-317567 produced no effects on MVP, CPP, RP, 1st PD, 2nd PD, BCD, and BCP (Figure 5).

**TABLE 2:** Cystometry variables in response to pH changes from 6.3 to 3.0.

	PT (mmHg)	MVP (mmHg)	CPP (mmHg)	RP (mmHg)	1st PD (s)	2nd PD (s)	BCD (s)	ICI (s)	BCP (μl/mmHg)
SAL (pH 6.3)	4.8 ± 0.2	24.7 ± 1.1	14.5 ± 1.8	1.1 ± 0.1	10.1 ± 0.9	15.6 ± 0.9	25.7 ± 0.6	392.1 ± 33.0	55.3 ± 4.2
AA (pH 3.0)	3.9 ± 0.2**	17.3 ± 1.2***	8.3 ± 0.8**	1.7 ± 0.1**	8.2 ± 1.2	10.8 ± 1.4**	19.0 ± 2.1**	76.0 ± 8.5****	16.5 ± 1.2****

Values are means ± SEM for 12 mice. Comparisons between SAL and AA by unpaired *t* test: **P < 0.01, ***P < 0.001, ****P < 0.0001. PT, pressure threshold for inducing micturition contraction; MVP, maximal voiding pressure; CPP, closing peak pressure; RP, resting pressure; 1st PD, 1st phase duration of bladder contraction; 2nd PD, 2nd phase duration of bladder contraction; BCD, bladder contraction duration; ICI, intercontraction interval; BCP, bladder compliance; SAL, saline infusion; AA, acetic acid infusion.

### Intravesical Perfusion of A-317567

**FIGURE 6:**
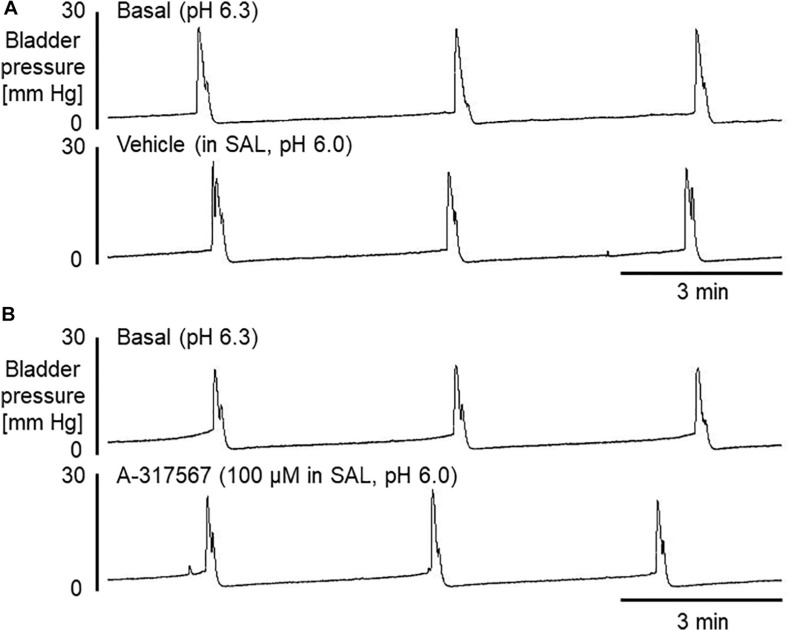
Bladder activity was induced in mice during continuous infusion cystometry (30 μl/min) with pH 6.3 saline followed by pH 6.0 saline containing vehicle **(A)** or A-317567 (100 μM) **(B)**. Intravesical perfusion of neither A-317567 nor vehicle produced effects on bladder activity. SAL, saline (pH 6.0) infusion.

**FIGURE 7:**
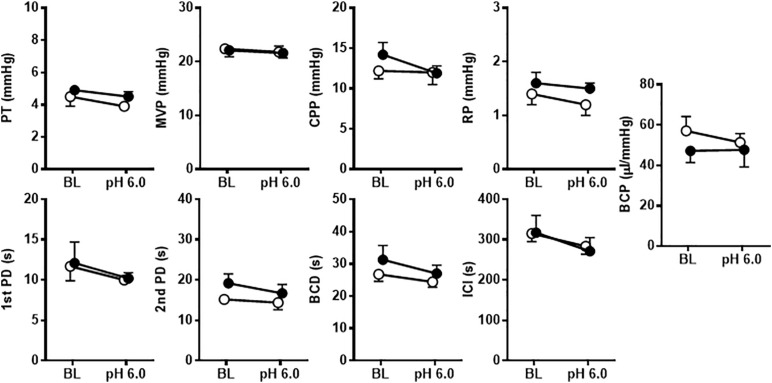
The effects of intravesical infusion of A-317567 (100 μM, •, *n* = 6) or vehicle (∘, *n* = 6) on bladder activity during saline (pH 6.0) infusion cystometry were evaluated according to the presented cystometry parameters. Statistical analysis by two-way repeated measures ANOVA revealed that, in comparison with vehicle, A-317567 had no effect on these cystometry variables. Furthermore, a drop of pH from 6.3 (BL) to 6.0 did not produce any changes in these cystometry variables for either group (*post hoc* Sidak’s multiple comparisons test). BL, baseline.

Bladder activity was not changed in response to a change in the pH of the intravesically infused solution from 6.3 to 6.0 (Figures 6, 7). Compared with vehicle perfusion, intravesical perfusion of A-317567 (100 μM in pH 6.0 saline) produced no changes in the cystometric variables (Figures 6, 7).

**FIGURE 8:**
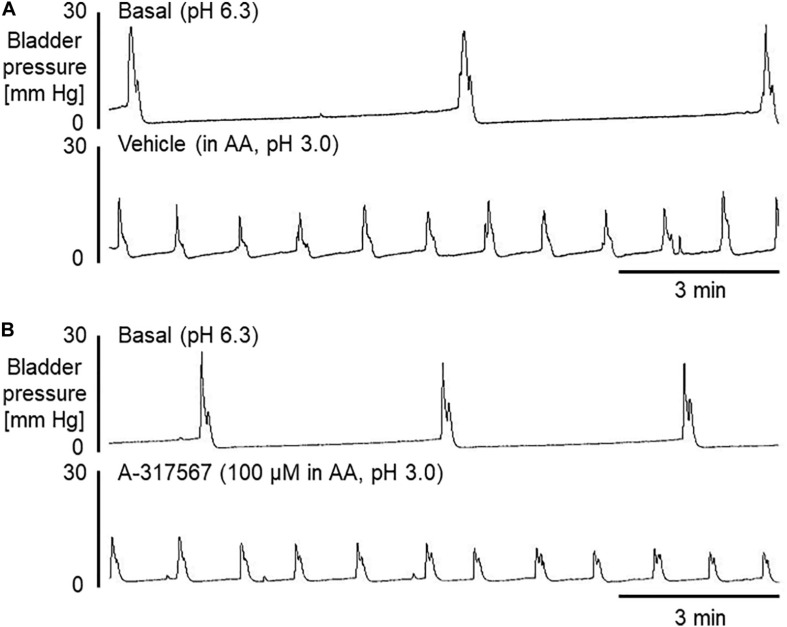
Bladder activity was induced in mice during continuous infusion cystometry (30 μl/min) with pH 6.3 saline followed by pH 3.0 acetic acid containing vehicle **(A)** or A-317567 (100 μM) **(B)**. Intravesical perfusion of A-317567 did not alleviate acetic acid-induced bladder hyperreflexia, showing no difference from vehicle’s effect.

**FIGURE 9:**
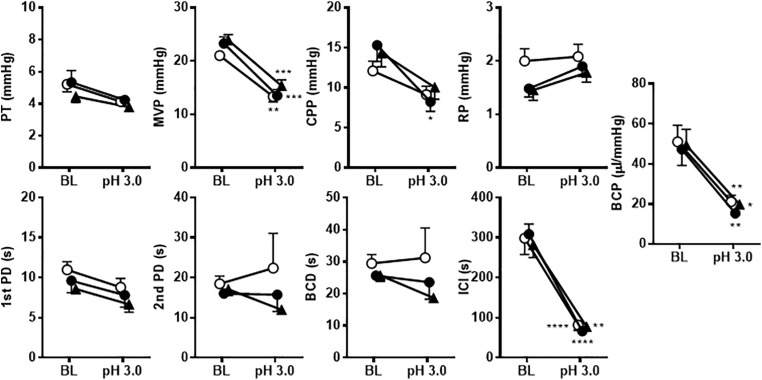
The effects of intravesical infusion of A-317567 (100 μM, •, *n* = 6; 1 mM, 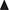, *n* = 5) or vehicle (∘, *n* = 6) on bladder activity during pH 3.0 acetic acid infusion cystometry were evaluated according to the presented cystometry parameters. Statistical analysis by two-way repeated measures ANOVA revealed that, in comparison with vehicle, A-317567 had no effect on these cystometry variables. The differences from BL (pH 6.3) to pH 3.0 in each treatment group are indicated as *P < 0.05; **P < 0.01; ***P < 0.001; and ****P < 0.0001 by *post hoc* Sidak’s multiple comparisons test. BL, baseline.

Intravesical perfusion with vehicle in pH 3.0 diluted acetic acid solution significantly decreased the MVP, ICI and BCP but did not affect other cystometric variables (Figures 8, 9). Compared with vehicle perfusion, intravesical perfusion of A-317567 at either 100 μM or 1 mM (cystometric recording not shown) did not change the cystometric variables that responded to intravesical irritation with pH 3.0 solution (Figures 8, 9).

## Discussion

The present study showed that i.p. administration of A-317567, a potent non-amiloride ASIC blocker, increases urinary bladder capacity during normal saline infusion cystometry and alleviates bladder hyperreflexia induced by intravesical infusion of acetic acid in decerebrated, unanesthetized mice. These results suggest that ASICs are involved in bladder mechanosensation and nociception. However, systemic injection of A-317567 neither changed the MVP during normal saline infusion cystometry nor reversed the reduction in MVP induced by intravesical acid irritation, thus suggesting that ASICs do not participate in efferent modulation of bladder contraction. The increase in the PT by i.p. injection of A-317567 was mainly attributable to the increase in the volume collected in the bladder before micturition during continuous infusion cystometry (Daly et al., 2007; Kira et al., 2017). However, intravesical perfusion of A-317567 produced no changes in urinary bladder activity during cystometry with or without acid irritation of the bladder, suggesting that ASICs in the urothelium do not play a significant role in the control of bladder activity.

Although quantitative analysis using RT-PCR in our previous study showed that the ASIC1, ASIC2 and ASIC3 genes are expressed in the mouse bladder mucosa (Kobayashi et al., 2009), the results of this study, in which *in vivo* cystometric evaluation was performed, did not prove that ASICs in the urothelium play significant roles in the control of bladder function in this species. However, possible contributions of rat urothelium ASICs to bladder activity have been previously examined in *in vivo* and *in vitro* experiments. A previous study (Kullmann et al., 2009) using patch-clamp and fura-2 Ca^2+^ imaging techniques investigated the functional properties of ASICs in cultured urothelial cells from rats. Transient Ca^2+^ responses were repeatably reduced by 75% in the presence of amiloride (10 μM), a non-selective ASIC blocker. Seventy-five percent of cultured urothelial cells showed responses to a pH change (pH 5.5) by acetic acid, exhibiting biphasic-like currents that were sensitive to amiloride (10 μM). The investigators claimed that these “neuron-like” properties mediated via ASIC activity may be involved in the release of transmitters that can act on afferent nerves or smooth muscle to modulate bladder responses. Another study (Corrow et al., 2010) showed that ASIC2a and ASIC3 protein expression was evident in the suburothelial plexus of normal rats, although it was minimal in the urothelium, and that chemical cystitis induced by cyclophosphamide led to upregulation of ASIC2a and ASIC3 protein expression in the urothelium and suburothelial plexus. These results imply the possibility of functional contributions of ASIC2a and ASIC3 in rat bladder mucosa to the normal micturition reflex and bladder inflammation-induced hyperreflexia. *In vivo* cystometric evaluation in rats (Du et al., 2007a) showed that intravesical perfusion of amiloride (1 mM) increased bladder intercontraction intervals, suggesting the possibility that epithelial sodium selective ion channels, including ASICs, are involved in sensory signal transduction during the modulation of bladder capacity. However, the interpretation of this result is complicated since the change produced by amiloride may be attributable to multiple types of channels other than ASICs [e.g., TRPV2 (Yu et al., 2011), TRPA1 (Du et al., 2007b), ENaC (Du et al., 2007a), T-type calcium channel (Tang et al., 1988)] as well as to the unspecified high concentration used. The cause of the difference in the response to ASIC blockers between rats and mice is unknown, and further investigations are thus necessary to determine the importance of urothelial ASICs in the modulation of urinary bladder function.

The bladder-filling volume necessary to induce micturition can be influenced by the compliance of bladder smooth muscle since smooth muscle tension correlates with bladder afferent output (Daly et al., 2007; Grundy et al., 2019). Furthermore, the majority of bladder sensory afferent nerves innervating the bladder are not located in the vicinity of the urothelium but are embedded within the bladder smooth muscle in mice (Spencer et al., 2018). These findings taken together with those of our previous study (Kobayashi et al., 2009) showing that ASIC genes and proteins are highly expressed in mouse detrusor muscle, suggest the possibility that ASICs in bladder smooth muscle may also contribute to changes in the bladder-filling volume. However, the present study showed that in comparison with vehicle, A-317567 decreased BCP during pH 6.3 saline infusion cystometry and produced no change in BCP related to bladder hyperreflexia induced by pH 3.0 acetic acid. During the early stage of the bladder-filling phase, the intravesical pressure increased approximately linearly as the bladder volume increased at a steady rate (Kira et al., 2017). However, after a certain volume was infused, BCP decreased exponentially as the infused volume in the bladder increased further (Figure 2) (Daly et al., 2007; Grundy et al., 2019). Moreover, the expansion of the bladder was restricted due to the intravesical catheter installed on the bladder wall. Thus, we presumed that the mechanism underlying the decreased BCP was a combination of these factors. Moreover, blockade of ASICs by A-317567 showed no or little effect on bladder smooth muscle activity during pH 3.0 acetic acid infusion cystometry compared with the effect of vehicle treatment, which suggested that the increased ICI (or increased bladder-filling volume) was not produced via bladder smooth muscle (Figure 4).

The present results revealed that the action site of A-317567 is located in the neural circuit that modulates reflex micturition but not the bladder. When administered systemically, A-317567 displayed extremely poor penetration into the central nervous system, suggesting that it mainly acts on the peripheral nervous system (Dubé et al., 2005). A large number of ASIC subunit genes (i.e., ASIC1, ASIC2, and ASIC3) have been found in the DRGs that innervate the urinary bladder (Kobayashi et al., 2009). The ASIC protein has been detected in the soma and in the peripheral nerve endings of DRG neurons (Lingueglia, 2007). Taking these results together, it seems reasonable to conclude that the site responsible for the action of the ASIC blocker is the DRGs. In a study using RT-PCR (Kobayashi et al., 2009), the gene expression of ASIC2 was the highest among the ASIC subunits in the lumbosacral DRGs (L6 and S1) that innervate the urinary bladder in mice. However, the gene expression levels of ASIC1 and ASIC3, which were detected almost equivalently, were one-fifth to one-fourth of those of the ASIC2 gene. Intriguingly, the gene expression levels of ASIC2 and ASIC3 in DRGs of female mice were markedly higher than those in DRGs of male mice, suggesting that there is a sex difference in proton-evoked signal transduction in bladder afferent pathways. Our previous studies (Yoshiyama et al., 2008, 2010) showed that in female mice, the bladder response to intravesical acetic acid irritation is more sensitive than that in male mice, which presents as marked bladder hyperreflexia. Thus, these results suggest that increased levels of ASIC2 and ASIC3 gene expression in DRGs may be involved in the vulnerability of the female bladder in response to acidic irritants.

Drug or vehicle was injected via an i.p. catheter in a small volume (300 μl/kg) at the right edge of the upper intra-abdominal region of the animals. Thus, it is less likely that the drug produced the results observed here by acting directly at the bladder wall and/or pelvic plexus through a mechanism involving sympathetic nerve modulation via the hypogastric nerves than by acting at another site, although we need to be careful when considering the possibility that the drug penetrated directly to action sites other than DRGs.

Another laboratory (Montalbetti et al., 2018) using mice lacking the ASIC3 gene previously showed results opposite to ours concerning the role of ASICs in controlling urinary bladder activity. Void spot assays showed that the voided volume per micturition in ASIC3-KO mice was smaller than that in their wild-type (WT) littermates under conscious conditions. However, cystometric evaluation under urethane-anesthetized conditions revealed that the voided volumes of ASIC3-KO and WT mice were similar. In addition to the compensatory effects resulting from gene knockout in the central as well as the peripheral nervous system, the discrepancies between our results and the other findings under conscious and urethane-anesthetized conditions may be attributable to other phenotypes caused by deletion of the ASIC3 gene and to the use of urethane anesthesia, respectively. First, the plasma level of testosterone in ASIC3-KO mice was markedly lower than that in WT mice (Burnes et al., 2008). ASIC3 is expressed in the testes, ovaries and adrenal glands (Babinski et al., 1999, 2000) and is associated with testosterone production and/or secretion in mammals. Testosterone regulates the expression of ion channels such as calcium channels in smooth muscle as well as skeletal and cardiac muscle (Bowles et al., 2004; Golden et al., 2004; Nudler et al., 2005; Michels et al., 2006). Compelling evidence from previous studies (Kwon et al., 2014; Hristov et al., 2016; Bonilla-Becerra et al., 2017) suggests that a low serum level of testosterone is significantly associated with instability/hyperactivity of the urinary bladder. Thus, it is probable that a low level of serum testosterone in conscious ASIC3-KO mice had a great impact on the urine storage phase, provoking detrusor excitability and reducing the voided volume. Second, previous studies revealed that mice lacking genes that are related to bladder afferent transmission respond differently to urethane than do WT mice, exhibiting inhibition or disruption of the micturition reflex (Kiss et al., 2001; Birder et al., 2002). Similarly, it is possible that a lack of ASIC3 in neural pathways that control bladder function interacts with the effect of urethane, leading to an alteration in urinary bladder activity.

In this study, the effects of A-317567 (30 μmol/kg, i.p.) lasted for only 10 to 15 min. The dose was selected according to a previous study assessing rat hind paw withdrawal latency to heat which showed an analgesic effect of A-317567 in Sprague-Dawley rats (Dubé et al., 2005). The study revealed that a dose of 30 μmol/kg (i.p.) given 30 min prior to testing exerted a significant effect on reducing chronically induced thermal hyperalgesia. The decreased duration of A-317567’s effects in our study compared to this previous study may be ascribed to the different evaluation targets (i.e., bladder function versus pain in the hind paw) and/or species differences (i.e., mice versus rats). Regarding the latter possibility, for example, the duration of the anesthetic effect of 1.2 g/kg (i.p.) urethane is 8 to 10 h in Sprague-Dawley rats, whereas it is only 40 min in C57BL/6 mice (undescribed observations from previous experiments performed by M. Yoshiyama: Yoshiyama et al., 1991; Yoshiyama et al., 1993a; Kiss et al., 2001). The difference between mice and rats in the duration of the effect of A-317567 as well as that of urethane is unknown, although we speculate that it is attributed to differences in distribution and metabolism between the two species.

In the present study, we showed that an ASIC blocker alleviated the hyperactive bladder response induced by acute intravesical infusion of acetic acid. In further developments for the treatment of IC/PS, it will be necessary to examine whether blockade of ASICs can exert beneficial effects on chronically or persistently induced bladder hyperreflexia and pain. Cyclophosphamide has often been used to induce persistent bladder inflammation. However, we need to pay considerable attention to the interpretation of the results of experiments using cyclophosphamide, especially when assessing neural pathways in both the peripheral and central nervous systems, because cyclophosphamide causes other adverse systemic symptoms including peripheral neuropathy (i.e., effects on nerves and/or DRGs) (Kemp et al., 1996; Tschöp et al., 2001; Grisold et al., 2012). To date, hydrogen peroxide (H_2_O_2_)-induced cystitis has been used to model long-lasting bladder pain and confers a persistent inflammatory profile lasting up to 14 days in mice and rats (Masuda et al., 2007; Yeh et al., 2010; Homan et al., 2013). It would be intriguing to investigate whether the roles of ASICs are preserved during persistent exposure of the urinary bladder to noxious stimuli using such an animal model and whether an ASIC blocker can ameliorate bladder symptoms.

## Conclusion

The present results showed that inhibition of afferent signal transduction via ASICs, presumably in the DRGs but not in the lower urinary tract (i.e., the urinary bladder and urethra), increased functional bladder capacity during normal saline infusion and ameliorated bladder hyperactivity induced by acidification in the bladder in mice. This study suggests that ASICs can be therapeutic targets for irritable bladder conditions such as IC/BPS as well as overactive bladder and that an ASIC blocker such as A-317567 may alleviate these lower urinary tract symptoms.

## Data Availability Statement

The datasets generated for this study are available on request to the corresponding author.

## Ethics Statement

The animal study was reviewed and approved by the University of Yamanashi Institutional Animal Care and Use Committee.

## Author Contributions

MY, HK, and IA contributed to conception and design of research. MY performed all the experiments, analyzed data, interpreted results of experiments, prepared figures, and wrote the manuscript. MY, HK, MT, and IA reviewed and approved final version of the manuscript. All authors contributed to the article and approved the submitted version.

## Conflict of Interest

The authors declare that the research was conducted in the absence of any commercial or financial relationships that could be construed as a potential conflict of interest.
