# Corrigendum: Blockade of Acid-Sensing Ion Channels Increases Urinary Bladder Capacity With or Without Intravesical Irritation in Mice

**DOI:** 10.3389/fphys.2020.624382

**Published:** 2020-12-15

**Authors:** Mitsuharu Yoshiyama, Hideki Kobayashi, Masayuki Takeda, Isao Araki

**Affiliations:** ^1^Department of Urology, Graduate School of Medicine, University of Yamanashi, Chuo, Japan; ^2^Shintotsuka Hospital, Yokohama, Japan; ^3^Kobayashi Urology Clinic, Kai, Japan; ^4^Kusatsu Public Health Center, Kusatsu, Japan

**Keywords:** A-317567, acetic acid, bladder pain syndrome, dorsal root ganglion, intravesical irritation, mechanosensation, nociception

In the original article, there was a mistake in the legend for Figure 9 as published. The symbol for 1 mM A-317567 is incorrect. The correct legend appears below.

**FIGURE 9** | “The effects of intravesical infusion of A-317567 (100 μM, •, *n* = 6; 1 mM, ▴, *n* = 5) or vehicle (◦, *n* = 6) on bladder activity during pH 3.0 acetic acid infusion cystometry were evaluated according to the presented cystometry parameters. Statistical analysis by two-way repeated measures ANOVA revealed that, in comparison with vehicle, A-317567 had no effect on these cystometry variables. The differences from BL (pH 6.3) to pH 3.0 in each treatment group are indicated as ^*^P < 0.05; ^**^P < 0.01; ^***^P < 0.001; and ^****^P < 0.0001 by *post hoc* Sidak's multiple comparisons test. BL, baseline.”

The authors apologize for this error and state that this does not change the scientific conclusions of the article in any way. The original article has been updated.

